# Predicting Future Respiratory Hospitalizations in Extremely Premature Neonates Using Transcriptomic Data and Machine Learning

**DOI:** 10.3390/children12080996

**Published:** 2025-07-29

**Authors:** Bryan G. McOmber, Lois Randolph, Patrick Lang, Przemko Kwinta, Jordan Kuiper, Kartikeya Makker, Khyzer B. Aziz, Alvaro Moreira

**Affiliations:** 1 Department of Pediatrics, University of Texas Health San Antonio, San Antonio, TX 78229, USA; bgmcomber@cmh.edu (B.G.M.); randolphl@uthscsa.edu (L.R.);; 2 Department of Pediatrics, Jagiellonian University Medical College, 30-663 Krakow, Poland; przemko.kwinta@uj.edu.pl; 3 Department of Environmental and Occupational Health, George Washington University, Washington, DC 20052, USA; jordan.kuiper@email.gwu.edu; 4 Department of Pediatrics, Johns Hopkins University, Baltimore, MD 21287, USA; kmakker1@jhmi.edu (K.M.);

**Keywords:** preterm infants, transcriptomics, respiratory morbidity, machine learning, bronchopulmonary dysplasia, bioinformatics

## Abstract

Background: Extremely premature neonates are at increased risk for respiratory complications, often resulting in recurrent hospitalizations during early childhood. Early identification of preterm infants at highest risk of respiratory hospitalizations could enable targeted preventive interventions. While clinical and demographic factors offer some prognostic value, integrating transcriptomic data may improve predictive accuracy. Objective: To determine whether early-life gene expression profiles can predict respiratory-related hospitalizations within the first four years of life in extremely preterm neonates. Methods: We conducted a retrospective cohort study of 58 neonates born at <32 weeks’ gestational age, using publicly available transcriptomic data from peripheral blood samples collected on days 5, 14, and 28 of life. Random forest models were trained to predict unplanned respiratory readmissions. Model performance was evaluated using sensitivity, specificity, positive predictive value, negative predictive value, and area under the receiver operating characteristic curve (AUC). Results: All three models, built using transcriptomic data from days 5, 14, and 28, demonstrated strong predictive performance (AUC = 0.90), though confidence intervals were wide due to small sample size. We identified 31 genes and eight biological pathways that were differentially expressed between preterm neonates with and without subsequent respiratory readmissions. Conclusions: Transcriptomic data from the neonatal period, combined with machine learning, accurately predicted respiratory-related rehospitalizations in extremely preterm neonates. The identified gene signatures offer insight into early biological disruptions that may predispose preterm neonates to chronic respiratory morbidity. Validation in larger, diverse cohorts is needed to support clinical translation.

## 1. Introduction

Extremely premature neonates, defined by the World Health Organization as neonates born before 28 weeks of gestation [1,2,3], are at higher risk for a wide range of short- and long-term health complications. Among these, respiratory morbidities are particularly prevalent and frequently result in recurrent hospitalizations during early childhood [4]. These readmissions are often attributed to underlying factors such as arrested lung development, poor lung function, heightened susceptibility to infections, and chronic respiratory conditions, including asthma and bronchopulmonary dysplasia [5,6]. Timely identification of neonates at greatest risk for future respiratory admissions is essential to improving outcomes.

Early identification, ideally within the first month of life, could enable clinicians to implement targeted preventive strategies, tailor individualized care plans, and allocate resources more efficiently for families. While demographic and clinical variables offer some prognostic value, they may be insufficient to fully capture the biological heterogeneity underlying respiratory outcomes in this population. Thus, reliance solely on traditional predictors such as bronchopulmonary dysplasia (BPD) may overlook critical early-life molecular signals associated with adverse outcomes.

To address these limitations, we explore the use of transcriptomic profiling, which captures genome-wide RNA expression patterns, as a means of identifying molecular signatures associated with poor respiratory trajectories [7]. When integrated with clinical and demographic data, transcriptomic analysis may reveal dysregulated pathways that predispose neonates to respiratory morbidity. Combining these multidimensional data sources using machine learning approaches may improve predictive accuracy by identifying complex, non-linear patterns associated with future hospitalization risk [8].

The central hypothesis of this study is that transcriptomic data collected within the first month of life, when combined with demographic information, can improve the prediction of long-term respiratory-related hospitalizations in very low gestational age neonates. This study aims to develop predictive models that integrate transcriptomic and demographic data collected within the first month of life to forecast respiratory-related hospitalizations in extremely premature neonates during their first four years.

## 2. Materials and Methods

### 2.1. Study Design and Participants

This retrospective cohort study analyzed transcriptomic and clinical data from 111 preterm neonates admitted to the neonatal intensive care unit (NICU) at University Children’s Hospital in Krakow, Poland, between September 2008 and November 2010 [9]. Of these, 58 neonates met the following inclusion criteria: (a) gestational age < 32 weeks, (b) birthweight < 1500 g, (c) requirement for respiratory support at time of inclusion, (d) availability of transcriptomic data at all predefined time points, and (e) complete clinical follow-up over four years post-discharge. Transcriptomic data were obtained from the publicly available Gene Expression Omnibus dataset GSE32472 and through personal communication with the principal investigator (Dr. Kwinta). This study adheres to the STROBE reporting guidelines for observational studies and the TRIPOD checklist for multivariable prediction model development and validation. Please refer to Supplementary Files S1 and S2.

### 2.2. Data Collection and Processing

This study is a secondary analysis of publicly available transcriptomic and clinical data obtained from the Gene Expression Omnibus (GEO) under accession number GSE32472. In the original study, peripheral blood samples were collected from preterm neonates on days 5, 14, and 28 of life, and whole-transcriptome profiling was performed using the Affymetrix GeneChip Human Gene 1.0 ST Arrays. Detailed RNA extraction, quality control, and microarray procedures are described in the original publication by Kwinta et al. [9].

For this analysis, raw probe-level expression data were processed using a two-step normalization pipeline. First, data were log2-transformed to stabilize variance. Second, quantile normalization was applied across arrays to ensure comparability. As all samples were processed within a single study under uniform laboratory conditions, no additional batch correction was applied.

Clinical data, including gestational age, birth weight, duration of mechanical ventilation, and oxygen therapy duration, were extracted from patient records in the parent study and through direct communication with the principal investigator. BPD was defined according to the 2001 NICHD criteria [10]. For this analysis, transcriptomic data (~22,000 genes per sample) were merged with clinical and demographic variables to create integrated datasets for each time point (days 5, 14, and 28). No missing clinical data were reported for the selected 58 neonates. As such, no imputation methods were applied.

### 2.3. Outcome Measures

The primary outcome was the occurrence of at least one respiratory-related hospitalization (e.g., bronchiolitis, pneumonia, respiratory failure, apnea requiring evaluation and support, severe upper respiratory infection requiring close observation) within the first four years of life. A respiratory-related hospitalization was defined as any unplanned hospital admission due to pulmonary complications following initial discharge from the neonatal intensive care unit.

### 2.4. Feature Selection

To identify candidate genes for predictive modeling, we performed univariate analysis using the Wilcoxon rank sum test via the R stats package, comparing gene expression between neonates who were and were not hospitalized for respiratory causes. Genes with a nominal *p*-value < 0.05 were retained for downstream modeling. This yielded 454 genes from the day 5 dataset, 722 from day 14, and 320 from day 28. However, none of these genes remained statistically significant after correction for multiple testing using the false discovery rate (FDR) method.

Given the limited sample size and the high dimensionality of the transcriptomic data, we adopted an exploratory, data-driven approach focused on predictive utility rather than statistical inference. While FDR is important for biomarker validation, it may exclude potentially relevant genes in early-phase discovery, justifying our use of nominal thresholds to identify predictive candidates. Therefore, genes meeting nominal significance thresholds were used as candidate features in supervised machine learning models to assess their utility in predicting respiratory-related hospitalizations.

To promote consistency and reduce overfitting, we focused on biological robustness and temporal consistency, identifying genes overlapping across multiple time points (day 5 and 14; day 14 and 28). This resulted in 31 shared genes, selected as core transcriptomic features.

### 2.5. Machine Learning Models and Analysis

We used the R caret package for model development, randomly partitioning 75% of the cohort into a training set and 25% into a testing set. Predictive features included both numerical and categorical variables, such as gestational age, birth weight, duration of mechanical ventilation, oxygen therapy, and BPD diagnosis.

Categorical predictors were one-hot encoded as needed. To address class imbalance in the training set, we applied the Synthetic Minority Over-sampling Technique (SMOTE) using the DMwR package. Additionally, we incorporated class weights during model training, assigning a weight of 0.5 to the majority class (no hospitalization) and 1.5 to the minority class (hospitalization). These weights reflected the approximate class distribution (~30% hospitalized) and were intended to reduce bias toward the majority outcome. We assessed their impact by comparing model performance metrics, including AUC, sensitivity, and specificity, with and without weighted loss functions during cross-validation.

We used a random forest classifier with 10-fold cross-validation, repeated 10 times, for model tuning. Hyperparameters tuned included the number of trees (ntree from 100 to 1000) and the number of variables tried at each split (mtry, ranging from 2 to 10), using grid search with the highest AUC on the validation fold as the selection criterion. Model performance was assessed using sensitivity, specificity, positive predictive value (PPV), negative predictive value (NPV), AUC, and balanced accuracy.

To minimize data leakage, all preprocessing (SMOTE, normalization, feature selection) was restricted to the training set before testing. No external dataset was available for independent validation. As a partial remedy, we used internal resampling via repeated cross-validation. We acknowledge this limitation and suggest that future work validate findings in an independent cohort or via bootstrapped estimates of model generalizability.

### 2.6. Temporal Expression Analysis

To investigate the dynamic behavior of gene expression over time in relation to respiratory outcomes, we used linear mixed-effects models (LMMs) with the lme4 and emmeans R packages. Each gene was modeled with fixed effects for postnatal day (days 5, 14, and 28), hospitalization status (yes/no), and their interaction term to capture differential expression trajectories between outcome groups. Subject ID was included as a random intercept to account for within-subject correlation due to repeated measures.

Estimated marginal means were computed from the fitted models to visualize average gene expression trends over time, stratified by hospitalization status. Statistical contrasts between time points and between groups were performed to identify genes with significantly divergent trajectories.

For pathway-level analysis, predefined gene sets were mapped to curated biological processes using publicly available pathway databases (e.g., Gene Ontology, KEGG). Expression data for each pathway were summarized by calculating the mean expression of genes within the set. LMMs were then applied to these pathway-level summaries using the same modeling framework as for individual genes, allowing us to examine whether groups of functionally related genes displayed coordinated, outcome-specific changes over time.

## 3. Results

### 3.1. Participant Characteristics

The cohort included 58 extremely preterm infants, of whom 23 (40%) experienced at least one respiratory-related hospitalization within the first four years of life. There were no statistically significant differences between rehospitalized and non-rehospitalized infants in gestational age, birth weight, sex, delivery mode, surfactant administration, SGA status, or BPD diagnosis. Infants who were rehospitalized had a longer average duration of oxygen therapy (56.2 vs. 39.3 days), though this difference was not statistically significant (*p* = 0.14). A flow diagram summarizing participant selection and exclusions is provided in Supplementary Figure S1.

### 3.2. Predictive Model Performance

We developed three predictive models based on transcriptomic profiles collected at days 5, 14, and 28 of life. Each model achieved strong discriminative performance, with an identical AUC of 0.90. However, confidence intervals varied slightly across time points:

- Day 5: AUC = 0.90, 95% CI: 0.73–1.00
- Day 14: AUC = 0.90, 95% CI: 0.78–1.00
- Day 28: AUC = 0.90, 95% CI: 0.54–1.00

(See Figure 1).

Clinical and demographic data for these infants can be found in Table 1. Model accuracy and class-specific performance varied by time point (Table 2). The model based on day 14 data achieved the highest balanced accuracy (0.84), followed by the day 5 model (0.74). The day 28 model had reduced specificity and the lowest balanced accuracy (0.64), suggesting limited discriminative capacity at later time points, despite similar AUCs.

Genes shown were selected based on consistent differential expression across at least two of three time points (days 5, 14, and 28) in relation to respiratory-related hospitalization within the first four years of life. Functional roles are based on relevant literature. The *p*-values represent unadjusted nominal significance from Wilcoxon rank-sum tests comparing gene expression between infants with and without respiratory-related hospitalizations. This gene list is exploratory and not adjusted for multiple comparisons due to the study’s focus on predictive model development in a limited sample size. See Kwinta et al. [9] for the original transcriptomic dataset (GEO accession: GSE32472).

### 3.3. Gene Expression and Pathway Analysis

#### 3.3.1. Day 5

**Figure 1:**
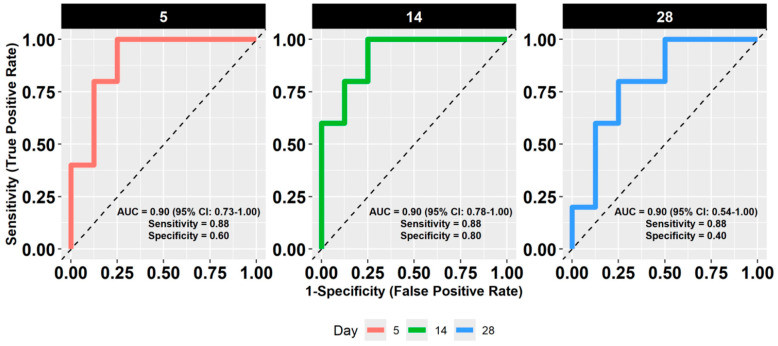
Receiver operating characteristic (ROC) curves for random forest models predicting respiratory-related hospitalizations in very low gestational age preterm infants using transcriptomic data collected on days 5 (red), 14 (green), and 28 (blue) of life. Each panel shows sensitivity versus 1-specificity for the corresponding time point. Model performance metrics are annotated within each panel, including area under the curve (AUC) with 95% confidence intervals, sensitivity, and specificity. N = 58: Outcome is defined as at least one respiratory-related hospitalization within the first four years of life.

**Figure 2:**
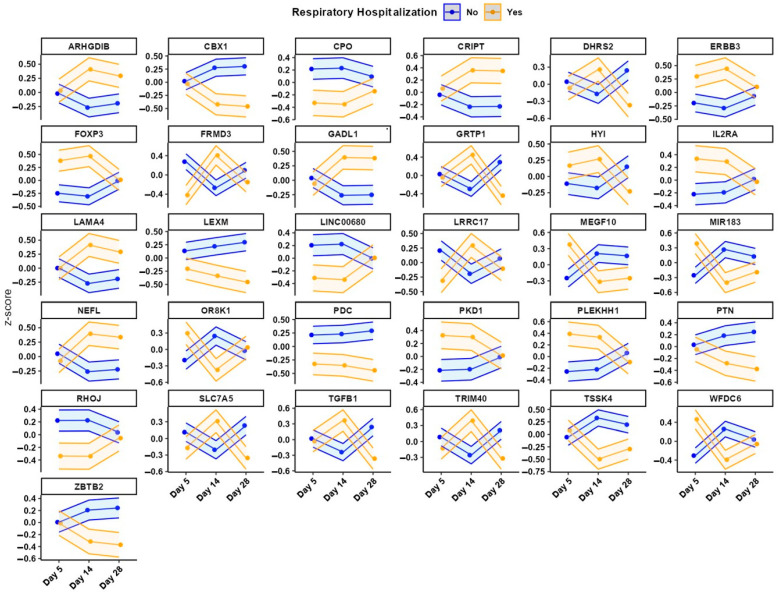
Longitudinal Expression Trajectories of 31 Predictive Genes Stratified by Respiratory-Related Hospitalization Status in Very Low Gestational Age Preterm Infants.

**Figure 3:**
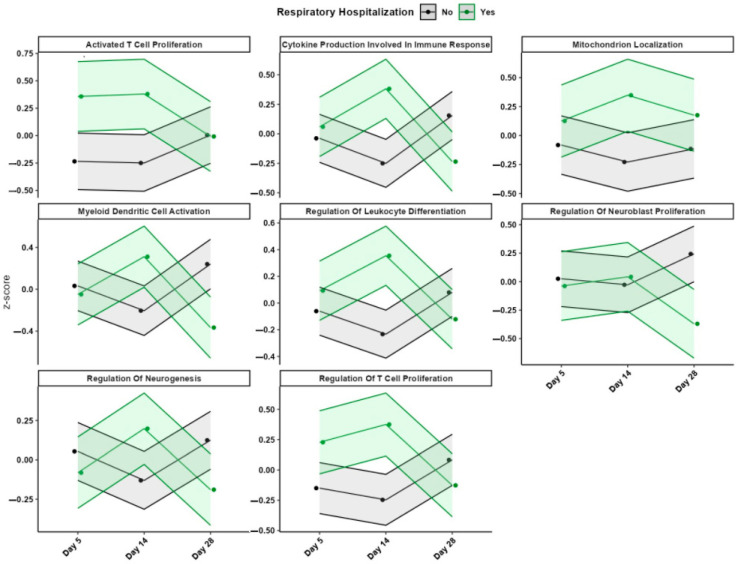
Longitudinal Expression Profiles of Selected Immune and Developmental Pathways Stratified by Respiratory-Related Hospitalization Status in Very Low Gestational Age Preterm Infants.

The model based on day 5 gene expression achieved moderate predictive performance (balanced accuracy = 0.74, AUC = 0.90; CI: 0.73–1.00; Figure 1, Table 2). Several genes were differentially expressed between neonates who experienced respiratory-related readmission and those who did not. A complete list of the 31 predictive genes, along with functional annotations and nominal *p*-values, is provided in Table 3. Longitudinal expression profiles for these genes are shown in Figure 2, stratified by hospitalization status. At this early time point, the pathway associated with activated T cell proliferation was significantly upregulated in the rehospitalized group (Figure 3). No other pathways showed statistically significant differential expression.

Each panel displays standardized gene expression (Z-scores) for one of the 31 genes used in predictive modeling, measured at days 5, 14, and 28 of life. Lines represent individual infants, color-coded by respiratory-related hospitalization status within the first four years: blue = no hospitalization; gold = at least one hospitalization. Genes were selected based on consistent presence across multiple time points and relevance to biological pathways implicated in respiratory morbidity. Expression patterns suggest early divergence in immune-related and developmental genes between outcome groups.

#### 3.3.2. Day 14

Transcriptomic data from day 14 yielded the highest predictive accuracy (balanced accuracy = 0.84; AUC = 0.90; Figure 1, Table 2). Several genes demonstrated directional changes in expression from day 5 to day 14, including FRMD3, which shifted from downregulated to upregulated in the rehospitalized group (Table 3, Figure 2). This time point also showed broader immune-related pathway activation. Specifically, pathways related to cytokine production involved in immune response, regulation of leukocyte differentiation, T cell proliferation, and mitochondrion localization were significantly upregulated in the rehospitalized infants (Figure 3). No pathways were significantly downregulated at this time point.

Line plots show standardized expression (Z-scores) of representative biological pathways across days 5, 14, and 28 of life. Pathways were selected based on differential activation patterns between infants who were rehospitalized (green lines) and those who were not (black lines) for respiratory causes within the first four years. Shaded areas reflect group-level variance. Several immune-related pathways (e.g., T cell proliferation, cytokine production) and developmental processes (e.g., neurogenesis, mitochondrion localization) exhibited distinct temporal trends between outcome groups.

#### 3.3.3. Day 28

Despite maintaining a high AUC (0.90), the day 28 model demonstrated the lowest balanced accuracy (0.64; Figure 1, Table 2). Gene-level results are shown in Figure 2, with corresponding annotations in Table 3. At the pathway level, no upregulated processes were identified in the rehospitalized group. However, two biological processes, myeloid dendritic cell activation and regulation of neuroblast proliferation, were significantly downregulated (Figure 3).

## 4. Discussion

### 4.1. Summary of Key Findings

In this study, we evaluated whether transcriptomic data from extremely preterm neonates, collected within the first month of life, could be used to predict respiratory-related rehospitalizations during early childhood. Using random forest models and expression profiles of 31 selected genes at days 5, 14, and 28, we achieved strong discriminative performance across all models (AUC = 0.90). The model based on day 14 data demonstrated the highest balanced accuracy, suggesting that this time point may offer optimal predictive resolution.

Interestingly, traditional clinical variables (e.g., gestational age, birth weight, duration of mechanical ventilation, and oxygen therapy) were not significantly associated with the primary outcome. Likewise, the presence or absence of BPD did not improve model performance. This finding contrasts with several studies that have identified BPD as a predictor of long-term respiratory morbidity [11,12]. However, it is consistent with other reports that question the reliability of BPD as a stand-alone prognostic marker [13,14]. Our findings suggest that transcriptomic biomarkers may capture underlying biological processes not reflected in traditional clinical parameters and could provide more precise risk stratification for respiratory outcomes in this high-risk population.

### 4.2. Interpretation of Results

Our findings support a growing recognition that molecular markers may offer improved risk stratification over traditional clinical predictors in extremely preterm neonates. While many prior studies have focused on BPD as a key prognostic indicator for long-term respiratory morbidity, this approach has shown inconsistent predictive performance. For example, Sun et al. examined long-term outcomes under two different diagnostic criteria for BPD and found significant variability in respiratory morbidity based on the definition used, suggesting that BPD alone may not be a stable or sufficient predictor of outcomes [15]. Similarly, Acuña-Cordero et al. reported that among preterm neonates with established BPD, the risk of hospitalization for acute lower respiratory infections was modulated by additional factors such as postnatal growth and comorbidities, not BPD severity alone [16]. In another study, Chen et al. observed that although BPD was associated with abnormal lung function trajectories during the first 36 months of life, substantial heterogeneity existed within BPD populations, with some neonates demonstrating relatively preserved lung function despite their diagnosis [17].

Our results build on this literature by demonstrating that transcriptomic profiles, collected within the first month of life, can identify the risk for respiratory-related hospitalizations even when traditional predictors, including BPD, are not informative. This finding suggests that underlying molecular dysregulation may be present early in life and may precede or act independently of the clinical phenotype of BPD.

Our study identified 31 genes differentially expressed at days 5, 14, and 28 of life in extremely preterm neonates. Unlike fixed clinical labels, gene expression conveys dynamic biological processes, including lung development, immune modulation, tissue remodeling, and metabolic stress, all of which are likely to influence respiratory vulnerability in nuanced and time-sensitive ways. For instance, early upregulation of immune-related genes may reflect heightened or dysregulated inflammatory responses that increase susceptibility to subsequent respiratory infections or inflammation-mediated lung injury. Persistent inflammation in the immature lung is a key driver of distorted lung development, impacting alveolar formation and contributing to the pathogenesis of BPD [18]. Conversely, downregulation of genes involved in tissue repair, cellular differentiation, or mitochondrial function may impair pulmonary development and compromise recovery following injury. Mitochondrial dysfunction has been shown to contribute to alveolar developmental arrest in models of BPD [19], and impaired mitochondrial bioenergetics is a fundamental mechanism underlying organ maturation failure in premature neonates [20].

Temporal variability in gene expression between neonates who were and were not rehospitalized within four years underscores the critical role of timing in gene regulation. The same gene may have different implications depending on when its expression is altered during postnatal development. Persistent upregulation of pro-inflammatory pathways, such as those involved in T-cell activation and proliferation between days 5 and 14, may indicate maladaptive immune activation. This sustained immune response could interfere with normal alveolarization or repair processes, increasing long-term respiratory vulnerability. Studies have shown that activated T lymphocytes, particularly IL-17A–producing γδ T cells, play a pivotal role in neonatal lung inflammation and the pathogenesis of BPD [21]. In contrast, for the rehospitalized group, the downregulation of genes related to neurodevelopment and mitochondrial activity at day 28 may reflect impaired cellular energetics or structural remodeling during a critical phase of lung maturation. Additionally, mitochondrial dysfunction has been implicated in the developmental failure of lungs in premature neonates, contributing to conditions like BPD [20].

## 5. Biological Insights from Gene Expression Patterns

### 5.1. Immune Regulation and Inflammation

Several genes in our panel modulate inflammatory responses, making immune dysregulation a hallmark of BPD pathogenesis. Interleukin-2 receptor alpha (IL2RA) is integral to T-cell activation and regulatory T-cell maintenance; dysregulation of IL2RA has been documented in asthma and chronic obstructive pulmonary disease (COPD) as part of the shared inflammatory cytokine network seen across chronic lung diseases [22,23]. Elevated IL2RA expression has been associated with heightened immune activation in preterm neonates, potentially contributing to the inflammatory milieu observed in BPD. Forkhead box P3 (FOXP3), essential for Treg cell development and function, has been implicated in BPD; studies have shown that increased frequencies of FOXP3+ Treg cells precede the development of BPD in preterm neonates, suggesting a compensatory response to ongoing inflammation. Transforming growth factor-beta 1 (TGFB1), a critical regulator of tissue remodeling and inflammation, plays a well-established role in airway fibrosis and remodeling and impaired alveolarization in BPD, asthma, and COPD [24,25].

### 5.2. Lung Development and Structural Integrity

Proper lung development is critical in preterm neonates, and disruptions can lead to BPD. Receptor tyrosine-protein kinase erbB-3 (ERBB3) is part of the epidermal growth factor receptor family and plays a role in epithelial cell differentiation. Dysregulation of ERBB3 signaling may impair alveolar development, contributing to the structural abnormalities seen in BPD [26]. Laminin subunit alpha-4 (LAMA4), a component of the extracellular matrix, is essential for basement membrane integrity and cell adhesion. Alterations in LAMA4 expression could affect alveolar structure and repair mechanisms, potentially influencing BPD progression [27]. Furthermore, neurofilament light chain (NEFL), while primarily associated with neuronal function, has been studied as a biomarker for neurodevelopmental outcomes in preterm neonates; elevated NEFL levels may reflect broader developmental disturbances that interplay with pulmonary development [28].

### 5.3. Metabolic and Oxidative Stress Responses

Metabolic processes and oxidative stress responses are integral to lung development and function. The enzyme dehydrogenase/reductase 2 (DHRS2) is involved in detoxification pathways and protection against oxidative stress, and in preterm neonates, impaired oxidative stress responses can exacerbate lung injury, leading to BPD [29]. The enzyme hydroxypyruvate isomerase (HYI) plays a role in glyoxylate metabolism, and disruptions in metabolic pathways can affect energy production and cellular repair mechanisms in the developing lung. The heterodimer solute carrier family 7 member 5 (SLC7A5) is involved in amino acid transport, which is essential for protein synthesis and cell growth; alterations in SLC7A5 expression may impact lung tissue development and repair. Collectively, these genes (and the proteins ultimately resulting from their expression) describe the importance of metabolic integrity in preventing BPD [30,31].

### 5.4. Limitations, Strengths, and Novel Contributions

Our study has several limitations. Most notably, the small sample size (n = 58) limits statistical power and generalizability and contributes to wide confidence intervals around performance metrics. Additionally, the observational nature of the study and lack of external validation restrict causal inference and raise questions about applicability in broader settings. The exploratory nature of the analysis, including the use of unadjusted *p*-values and a single-center cohort, further limits reproducibility. While our models performed well in this cohort, their performance in other populations remains unknown. Furthermore, we focused exclusively on transcriptomic data; other molecular layers such as proteomics, metabolomics, and epigenomics may provide complementary insights and improve predictive accuracy.

Importantly, the primary outcome was limited to the presence or absence of at least one respiratory-related hospitalization; data on the number, timing, or severity of hospitalizations were not available. Additionally, BPD was classified using legacy criteria from 2001, and not the more recent Jensen classification, which may impact the accuracy of BPD diagnosis and its prognostic implications. Lastly, death was not included as an outcome or competing event, which may have biased the observed associations if mortality disproportionately affected high-risk infants who may otherwise have experienced rehospitalizations.

Beyond methodological limitations, important practical barriers to clinical implementation must also be acknowledged. RNA sequencing remains expensive and is not currently integrated into routine neonatal care. Sample collection in extremely preterm infants is further complicated by limited blood volume, especially across serial time points. Additionally, the lack of bioinformatics infrastructure and EHR integration in many clinical environments limits the feasibility of deploying transcriptomic tools in real time. Most critically, the interpretability and clinical actionability of transcriptomic predictions remain limited, which may hinder clinician adoption unless paired with targeted interventions. Regulatory considerations and the need for validated, simplified assays also pose additional challenges to translation.

Despite these limitations, the study introduces several novel contributions. First, we demonstrate that transcriptomic data collected within the first month of life can meaningfully predict respiratory-related hospitalizations, an outcome of significant clinical importance. Second, we show that this predictive signal is present at multiple neonatal time points, suggesting that clinically actionable windows may extend across the first month of life. Third, we identify a specific panel of 31 genes and several associated pathways, which may offer biological insight into respiratory vulnerability and serve as candidate biomarkers for future investigation.

### 5.5. Next Steps for Translational Research and External Validation

Future studies should prioritize external validation using larger, multi-center cohorts to assess the generalizability of these models. Prospective longitudinal designs linking early transcriptomic profiles with long-term respiratory outcomes, including lung function testing, frequency and severity of hospitalizations, and health-related quality of life, are needed. Integration with multi-omic data (e.g., proteomics, epigenomics, and microbiome data) may improve predictive accuracy and biological insight. Developing simplified, clinically deployable transcriptomic tools (e.g., PCR panels) could support translational efforts. Finally, clinical decision-support systems that incorporate these molecular signatures will be critical for real-world implementation and must be co-developed with clinicians and informatics specialists.

## 6. Conclusions

This study demonstrates that transcriptomic profiling within the first month of life, when combined with machine learning, can effectively predict respiratory-related hospitalizations in extremely preterm neonates. Models built from gene expression data on days 5, 14, and 28 all demonstrated strong discriminative performance, with an AUC of 0.90 across time points. Notably, traditional clinical predictors, including gestational age, duration of respiratory support, and BPD diagnosis, were not significantly associated with the primary outcome, emphasizing the added value of molecular data. The identification of 31 dysregulated genes and their association with immune, developmental, and metabolic pathways offers new insight into the pathogenesis of respiratory morbidity in this vulnerable population.

These findings are exploratory and based on a small, single-center cohort without external validation. As such, they should be interpreted with caution. Further validation in larger and more diverse populations is essential to confirm the predictive utility of these findings. In parallel, translational efforts should focus on adapting transcriptomic-based models into clinically actionable, user-friendly formats that can be implemented within neonatal care workflows.

Ultimately, the integration of molecular prediction tools into neonatal care will require not only technical validation but also interdisciplinary collaboration, infrastructure development, and clinical acceptance. This study offers a preliminary step toward personalized risk stratification and targeted intervention in preterm respiratory disease.

## Figures and Tables

**Table 1:** Baseline Clinical and Demographic Characteristics of Very Low Gestational Age Preterm Infants, Stratified by Respiratory-Related Hospitalization Status Within the First Four Years of Life.

Characteristic	Overall N = 58	No Hospitalization N = 35	Yes Hospitalization N = 23	*p*-Value^2^
Gestational age (weeks)	27.7 (2.4)	27.7 (2.6)	27.9 (2.0)	0.8
Birth weight (g)	1010.6 (279.7)	999.1 (303.4)	1028.0 (244.8)	0.7
Sex:				0.6
Male	30 (52%)	17 (49%)	13 (57%)	
Female	28 (48%)	18 (51%)	10 (43%)	
Delivery mode:				0.5
Vaginal	20 (34%)	11 (31%)	9 (39%)	
Cesarean	38 (66%)	24 (69%)	14 (61%)	
Received surfactant	35 (60%)	20 (57%)	15 (65%)	0.5
SGA	10 (17%)	5 (14%)	5 (22%)	0.5
BPD status:				0.7
no BPD	22 (38%)	14 (40%)	8 (35%)	
BPD	36 (62%)	21 (60%)	15 (65%)	
Days on oxygen	46.0 (35.3)	39.3 (29.8)	56.2 (40.9)	0.14

Values are presented as mean (standard deviation) for continuous variables and n (%) for categorical variables. The *p*-values reflect comparisons between hospitalized and non-hospitalized groups using the Wilcoxon rank sum test for continuous variables, Pearson’s Chi-squared test for categorical variables, and Fisher’s exact test where expected cell counts were small. SGA = small for gestational age; BPD = bronchopulmonary dysplasia (defined as oxygen and/or positive pressure ventilation at 28 days of life).

**Table 2:** Genes Predictive of Respiratory-Related Hospitalization and Consistently Identified Across Days 5, 14, and 28 in Extremely Preterm Neonates, with Functional Annotations and Nominal *p*-Values.

Gene	Full Name	Function	*p* Value
*IL2RA*	Interleukin 2 Receptor Subunit Alpha	T-cell activation; immune regulation (inflammatory pathways implicated in BPD)	0.023152
*OR8K1*	Olfactory Receptor Family 8 Subfamily K Member 1	Olfaction	0.026595
*ERBB3*	Receptor Tyrosine-Protein Kinase erbB-3	Epithelial cell growth, lung development, and signaling	0.047818
*RHOJ*	Rho-related GTP-binding protein RhoJ	Endothelial cell migration, cytoskeletal organization; may relate to angiogenesis in BPD	0.024168
*PLEKHH1*	Pleckstrin Homology, MyTH4 and FERM Domain Containing H1	Poorly characterized; may have cytoskeletal roles	0.023152
*PKD1*	Polycystin 1	Cell-cell adhesion, mechanosensing	0.029837
*CPO*	Carboxypeptidase O	Proteolysis; potential involvement in epithelial turnover or lung matrix processing	0.035191
*WFDC6*	WAP-type four-disulfidr core (WFDC) domain family	Protease inhibition	0.00085
*MEGF10*	Multiple EFG Domains 10	Clearance of apoptotic cells; may influence alveolar macrophage function in lung injury resolution	0.031096
*LRRC17*	Leucine Rich Rpeat Containing 17	Bone marrow development	0.008806
*MIR183*	mir-183; microRNA	Post-transcriptional regulation	0.018601
*FRMD3*	FERM Domain Containing 3	Actomyosin structure organization	0.011754
*FOXP3*	Forkhead box P3	Regulatory T-cell differentiation; central to immune tolerance and inflammation	0.011757
*LINC00680*	Long non-coding RNA	Non-coding RNA	0.016033
*LEXM*	Lymphocyte Expansion Molecule	Unknown	0.00199
*HYI*	Hydroxypyruvate Isomerase	Glyoxyate metabolism	0.036595
*PDC*	Phosducin	Vision	0.006564
*ARHGDIB*	Rho GDP Dissociation Inhibitor Beta	Regulates Rho GTPase signaling; involved in cytoskeletal dynamics and immune cell trafficking	0.032516
*GRTP1*	Growth Hormone Regulated TBC Protein 1	GTPase activation	0.020734
*DHRS2*	Dehydrogenase/reducatse 2	Metabolism, detoxification, and protection from oxidative stress	0.046926
*TSSK4*	Testis-specific serine/threonine kinase family	Signal transduction	0.049636
*SLC7A5*	Solute carrier family 7 member 5	Amino acid transporter; important for cell growth and metabolism	0.024499
*CBX1*	Chromobox 1	Transcription regulation	0.005818
*TGFB1*	Transforming growth factor beta 1	Key regulator of fibrosis, alveolarization, and immune modulation in BPD	0.026595
*CRIPT*	CXXC Repeat Containing Interactor of PDZ3 Domain	Microtubule organization	0.039546
*GADL1*	Glutamate decarboxylase like 1	Amino acid metabolism	0.021173
*TRIM40*	Tripartite motif containing 40	Innate immunity and NF-κB suppression; may modulate inflammation	0.047818
*LAMA4*	Laminin subunit alpha 4	Component of basement membrane; implicated in vascular development and tissue remodeling	0.034501
*ZBTB2*	Zinc finger and BTB domain containing 2	Transcription regulation	0.00554
*PTN*	Pleiotrophin	Mitogen involved in angiogenesis and tissue repair; relevant to lung development	0.022548
*NEFL*	Neurofilament light chain	Neurofilament bundle assembly	0.045189

Gene function descriptions were obtained from GeneCards^®^: The Human Gene Database (www.genecards.org), accessed 1 July 2025. Functional relevance was interpreted in the context of known biological processes and literature, where applicable.

**Table 3:** Performance Metrics of Random Forest Models Predicting Respiratory-Related Hospitalizations Using Transcriptomic Data from Days 5, 14, and 28 in Very Low Gestational Age Preterm Infants.

Metric	Day 5	Day 14	Day 28
AUC	0.90	0.90	0.90
Confidence Interval	0.73–1.00	0.78–1.00	0.54–1.00
Sensitivity	0.88	0.88	0.88
Specificity	0.60	0.80	0.40
PPV	0.77	0.88	0.70
NPV	0.75	0.80	0.66
Balanced Accuracy	0.74	0.84	0.64

Metrics shown include area under the ROC curve (AUC), 95% confidence intervals (CI), sensitivity, specificity, positive predictive value (PPV), negative predictive value (NPV), and balanced accuracy. Balanced accuracy is calculated as the average of sensitivity and specificity.

## Data Availability

The genomic data presented in this study are available in Gene Expression Omnibus dataset GSE32472. Clinical and demographic data are available on request from the corresponding author, as these data are not publicly available.

## Supplementary Materials

The following supporting information can be downloaded at: https://www.mdpi.com/article/10.3390/children12080996/s1, Figure S1: Flow Diagram; File S1: STROBE Statement—checklist of items that should be included in reports of observational studies; File S2: Tripod-AI Statement—checklist completed in accordance with TRIPOD-AI guidelines to ensure transparent reporting of the prediction model. References [32,33] are cited in the supplementary materials.
